# Once again, the highlight of this edition (November-December 2026) of International Brazilian Journal of Urology is robotic surgery

**DOI:** 10.1590/S1677-5538.IBJU.2026.06.01

**Published:** 2026-09-21

**Affiliations:** 1 Universidade do Estado do Rio de Janeiro nidade de Pesquisa Urogenital Rio de Janeiro RJ Brasil Unidade de Pesquisa Urogenital - Universidade do Estado do Rio de Janeiro - Uerj, Rio de Janeiro, RJ, Brasil; 2 Hospital Federal da Lagoa Serviço de Urologia Rio de Janeiro RJ Brasil Serviço de Urologia, Hospital Federal da Lagoa, Rio de Janeiro, RJ, Brasil

The November-December number of International Brazilian Journal of Urology presents original contributions with a lot of interesting papers in different fields: Prostate cancer, Robotic Surgery, Kidney cancer, UPJ obstruction, Infertility, Bladder Cancer, Cryptorchidism, Endourology and Reconstructive Urology. The papers came from many different countries such as Brazil, China, Israel, Indonesia, Turkey and Portugal, and as usual the editor ´s comment highlights some of them. The editor in chief would like to highlight the work about Robot surgery. Robotic surgery in the last 5 years is responsible for more than 10% of submissions and 15% of accepted articles in International Brazilian Journal of Urology (1-7) and in this number more than 50% of the paper are about robotic surgery showing the importance of this technique in urology. We will comment below on a paper about radical prostatectomy that is the cover of this edition.

Dr. Lin and collegues from China, presented in id e20260376 (8) an interesting study about the preoperative urethral curve and this use to predict immediate urinary continence (UC) following Retzius-sparing robot-assisted radical prostatectomy (RS-RARP). The authors studied 580 patients in a multicenter study and concluded that this study proposes a novel quantitative method for assessing urethral curvature and validates its reliability in predicting immediate UC recovery following RS-RARP. This method is simple, reproducible, and highly feasible for implementation in routine clinical practice. This model provides a valuable quantitative tool for preoperative risk stratification and personalized surgical planning.

The Editor-in-chief expects everyone to enjoy reading.
